# Evaluating the use of silicone wristbands and urinary biomarkers to assess personal exposure to phthalates

**DOI:** 10.1038/s41370-026-00899-y

**Published:** 2026-04-28

**Authors:** Alana J. Ferris, Kylie W. Riley, Lehyla Calero, Darrell Holmes, Catherine Tobon, Matthew Gutierrez, Julianne Cook Botelho, Antonia M. Calafat, Maya Deyssenroth, Kim A. Anderson, Julie B. Herbstman

**Affiliations:** 1 Department of Environmental Health Sciences, Mailman School of Public Health, Columbia University, New York City, NY, USA; 2 Columbia Center for Children’s Environmental Health, Department of Environmental Health Sciences, Mailman School of Public Health, Columbia University, New York City, NY, USA; 3 Division of Laboratory Sciences, Centers for Disease Control and Prevention, Atlanta, GA, USA; 4 Environmental and Molecular Toxicology, Food Safety and Environmental Stewardship Program, Oregon State University, Corvallis, OR, USA

## Abstract

**BACKGROUND:**

Biomonitoring studies for phthalates often rely on concentrations of urinary biomarkers, but there is interest in broadening exposure assessment methods, especially for use with vulnerable populations like pregnant women. Silicone wristbands (wristbands) are non-invasive passive sampling devices that have been shown as valid exposure assessment tools for a variety of chemicals and could provide a complementary method of phthalate exposure assessment.

**OBJECTIVE:**

This study examined the relationship between phthalates detected in wristbands and their corresponding urinary metabolites to understand the ability of wristbands to capture phthalates.

**METHODS:**

This pilot study included 27 pregnant women from the New York City-based longitudinal birth cohort study, the Fair Start Cohort. One wristband and spot urine samples provided at three time points were collected during a single 48-hour period. Six phthalate levels in wristbands were compared with the corresponding 12 urinary metabolite concentrations. Linear regressions and k-means clustering were employed to describe the relationship between, and information generated from wristband and urine matrices.

**RESULTS:**

Three of the six parent phthalates were significantly positively associated with at least one of their metabolites in urine (butylbenzyl phthalate with monobenzyl phthalate; di-2-ethylhexyl phthalate with mono-2-ethylhexyl phthalate; di-iso-butyl phthalate with mono-hydroxy-isobutyl phthalate, mono-isobutyl phthalate, and the molar sum of these metabolites). Exposure marker profiles differed between wristband and urine matrices, which may reflect differences in routes of exposures.

**IMPACT:**

- This study demonstrates the ability of wristbands to capture personal exposure to phthalates and their substitutes. Additionally, using wristbands as an exposure assessment method could fill the gap in our understanding of the contribution of inhalation and dermal exposure routes for phthalate exposure, as these remain understudied in the literature.

## INTRODUCTION

Almost everyone is exposed to ortho-phthalates, also known as phthalates, through items packaged in plastic (e.g., foodstuffs and personal care products), making their presence ubiquitous in U.S. populations. Some phthalates are used as plasticizers added to polyvinyl chloride (PVC) plastics to make them more durable; others are commonly used as fragrance carriers in personal care products [1, 2]. Diethyl phthalate (DEP), di-n-butyl phthalate (DnBP), and di-iso-butyl phthalate (DiBP) are examples of low-molecular-weight (LMW) phthalates primarily used in cosmetics and personal care products. Di-2-ethylhexyl phthalate (DEHP), butylbenzyl phthalate (BBzP), diisononyl phthalate, and diisodecyl phthalate are high molecular weight (HMW) phthalates primarily used in PVC-based applications, plastics, and food packaging [3]. Because of growing concerns regarding phthalates’ effects on health, the US and other nations have banned or placed restrictions on some phthalates, prompting the use of other phthalates (e.g., di-2-ethylhexyl terephthalate (DEHTP)) and the development of phthalate substitutes, such as di(isononyl) cyclohexane-1,2-dicarboxylate (DINCH) [1, 4, 5]. We do not yet fully understand the extent of exposure to DINCH and DEHTP and the related health effects [6, 7]. Thus, there is a need to assess exposure to both phthalates and their substitutes to understand how human exposure is changing and their potential impacts on health.

Most biomonitoring studies of phthalates rely on human urine samples [8, 9], but there is interest in broadening methods of exposure assessment, especially for use with vulnerable populations, including pregnant women. Silicone wristbands (wristbands) are non-invasive passive sampling devices used to assess personal exposure to environmental contaminants and can provide a complementary method for phthalate exposure assessment to urine testing. The porous membrane of the wristbands sequesters the bioavailable fraction of chemicals from the air surrounding the wearer and through direct contact of the sampler with the skin or other surfaces [10–12]. These chemicals can then be extracted from the wristbands later. While urine is minimally invasive to collect, phthalates are rapidly metabolized and excreted [13], and exposures are likely episodic in nature. Phthalate metabolite concentrations in a single urine sample thus reflect recent exposure to these chemicals and may not adequately reflect long-term exposure. Instead, wristbands are lightweight and noninvasive, allowing them to be worn to capture aggregate exposure to phthalates over longer periods of time than a urine sample. Wristbands could, therefore, complement urine as an exposure assessment tool because of their noninvasive nature, low level of inconvenience, and ability to reflect exposure over longer periods [14].

Wristbands have been shown to capture a variety of chemicals in previous studies, including pesticides, polycyclic aromatic hydrocarbons (PAHs), and flame retardants [10, 15–18]. However, there is limited evidence demonstrating their use as a valid exposure assessment tool specifically for phthalates and their substitutes [19–22], and only two studies have compared wristbands and urine as phthalate exposure assessment tools in non-occupational settings [20, 23].

In this pilot study, we assessed personal exposure to phthalates and substitutes by measuring concentrations of these chemicals or their metabolites in matched wristband and urine samples. We aimed to examine if the phthalates and substitutes captured by wristbands and urine are similar to understand how wristbands may provide additional or complementary exposure information to urine.

## MATERIALS AND METHODS

### Study population and design

This study leverages an ongoing, longitudinal birth cohort study, the Fair Start Cohort, recruited at the Columbia Center for Children’s Environmental Health (CCCEH) in New York City, USA. We obtained informed consent from study participants in accordance with the Columbia University Institutional Review Board (IRB: AAAK6753). Participants ranged in age from 18 to 42 years, and 92% of the women were of Dominican origin (Table 1). Between 2018 and 2019, 27 women in their third trimester of pregnancy each wore one wristband for 48 hours. Study participants were instructed not to remove their wristband for the entire 48-hour sampling period. They were also instructed not to place lotion or other personal care products directly on the wristbands. Wristbands were collected year-round (See Supplementary Material, Table S1). Urine samples were collected from each woman at three time points (no first-morning voids) during the same 48-hour period in polypropylene specimen containers with caps (VWR International, Radnor, Pennsylvania, USA). Urine was sampled at the beginning of the sampling period, when the wristband was deployed, 24 and 48 hours after the sampling period began. All participants provided urine samples at all three time points. Samples were refrigerated before they were picked up at the end of the sampling period. Before they were sent for analysis at the Centers for Disease Control and Prevention (CDC), a sample pool from each participant was created by combining equal parts from each of the three urine samples. Using a pooled urine sample rather than individual spot samples can decrease measurement error, especially for biomarkers like phthalates that display within-subject variability because they are rapidly metabolized and excreted [24, 25]. Specific gravity of the pooled sample was measured with a digital handheld refractometer, and the pooled specific gravity was used to correct phthalate biomarker concentrations [23, 26]. Pooled urine samples were frozen (−80 °C) at the CCCEH and then shipped overnight frozen on dry ice to the CDC for analyses.

### Wristband methodology

#### Preparation and deployment.

Wristbands (including those used for quality control purposes) were purchased as a single batch from 24hour-wristbands.com (Houston, Texas, US). To remove any existing chemicals in the wristbands remaining after the manufacturing process, we followed the process described in Dixon et al., 2022 [27]. Briefly, wristbands were rinsed with deionized water and placed in a vacuum oven at Oregon State University (OSU) (300°C for 12 h at 0.1 Torr; Blue M vacuum oven, no. POM18VC; Welch^®^ DuoSeal pump, no. 1405, Mt. Prospect, Illinois, USA). To ship the wristbands to the CCCEH, wristbands were packed individually in airtight polytetrafluoroethylene (PTFE) bags (Welch Fluorocarbon, Dover, New Hampshire, US). After the 48-hour deployment period, wristbands were returned to the PTFE bags, sealed, and mailed back to OSU.

#### Cleaning and extraction.

To clean and extract the phthalates of interest from the silicone wristbands, we followed the process described in Adams et al. [11]. Briefly, wristbands were washed twice with 18 MΩ cm water and once with isopropanol to remove particles on the silicone surface so that extraction was restricted to phthalates contained within the wristbands, representing the fraction available to be absorbed into the body [16, 23]. To extract the phthalates of interest from the cleaned silicone, two separate 50 mL volumes of ethyl acetate at room temperature were added to the wristbands and then concentrated to 1 mL under nitrogen (Turbo-Vap L, Biotage, Charlotte, North Carolina, US; RapidVap, LabConco, Kansas City, Missouri, US; N-EVAP 111, Organomation Associates, Berlin, Massachusetts, US). Prior to analysis, wristband extracts were further cleaned with solid phase extraction using primary-secondary amine (PSA) cartridges.

#### Quantification.

Deuterated diamyl phthalate, di-n-butyl phthalate, and di-n-nonyl phthalate were added as internal standards to each wristband extract prior to analysis. Wristband extracts were analyzed for 31 phthalates and their substitutes with an Agilent 7890 A gas chromatograph (GC) paired with an Agilent 5975 C mass spectrometer (MS) detector with a DB-5MS column (Agilent) [10, 17]. The instrument parameters are detailed in Adams et al. [11]. The limits of detection (LODs) for all 31 phthalates and their substitutes ranged from 0.01 to 0.12 μg/g wristband (calculated using a wristband mass of 5.71 g). The list of target chemicals and LODs are in Table S2 on the μg/g scale for all 31 chemicals, respectively. Table 2 lists the six parent phthalates included in this study: DEHP, DEHTP, BBzP, DnBP, DEP, and DiBP.

#### Wristbands quality control summary.

Several QC samples were created and analyzed to account for potential chemical contamination during preparation, transport, and laboratory processing. Briefly, blank wristbands that traveled to and from study locations in the airtight PTFE bags were collected from each group of prepared wristbands and were analyzed using GC–MS. Solvent extraction blanks were collected and analyzed by performing the extraction process without wristbands. Laboratory-processed blank wristbands that underwent all laboratory processes (i.e., cleaning, extraction and instrument analysis) were also collected and analyzed. During phthalate quantification, instrument blanks and calibration verifications (CVs) were analyzed before and after approximately every fifteen samples to ensure the method met data quality objectives. Instrument blanks were GC vials filled with either hexane or ethyl acetate. All target chemical concentrations were below the LODs for the instrument blanks. Detected concentrations in the quality control samples were averaged and subtracted from sample concentrations. Surrogate recoveries averaged 99% across the study.

### Urine sample methodology

#### Phthalate metabolite quantification.

The analytical approach to measure 16 biomarkers of ortho- and non-ortho phthalates used a modification of the method reported in Silva et al., 2013 [28]. The target biomarkers were: monoethyl phthalate (MEP), mono-n-butyl phthalate (MBP), mono-2-hydroxy-n-butyl phthalate (MHBP), mono-isobutyl phthalate (MiBP), mono-2-hydroxyisobutyl phthalate (MHiBP), monobenzyl phthalate (MBzP), mono-3-carboxypropyl phthalate (MCPP), mono-2-ethylhexyl phthalate (MEHP), mono-2-ethyl-5-carboxypentyl phthalate (MECPP), mono-2-ethyl- 5-hydroxyhexyl phthalate (MEHHP), mono-2-ethyl-5-oxohexyl phthalate (MEOHP), mono carboxyisooctyl phthalates (MCOP), monooxononyl phthalates (MONP), mono carboxyisononyl phthalates (MCNP), mono-2-ethyl-5-carboxypentyl terephthalate (MECPTP), and mono-2-ethyl-5-hydrohexyl terephthalate (MEHHTP). MECPTP and MEHHTP were selected as the biomarkers of DEHTP exposure because they represent about 15% of the oral dose of DEHTP excreted in urine, whereas the other metabolites represent <1%, so may not be as useful exposure biomarkers [29, 30]. The 12 biomarkers included in this study are listed in Table 3.

Briefly, 0.1 mL of urine was spiked with an internal standard solution containing deuterium- or ^13^C-labeled analogs of the target metabolites and a buffered *β*-glucuronidase solution (*E. coli*-K12; 25 μL, pH 6.5/1 M), and incubated for a minimum of 120 min. The target analytes in the spiked urine were extracted using online solid phase extraction, chromatographically resolved by high-performance liquid chromatography, and detected using negative ion electrospray-ionization tandem mass spectrometry using a ThermoFinnigan TSQ Vantage triple quadrupole mass spectrometer equipped with an electrospray ionization interface while using multiple reaction monitoring mode. The LODs for all 16 target phthalate metabolites are listed in Table S3.

The CDC laboratory complies with the Clinical Laboratory Improvement Amendments of 1988 (CLIA) and is recertified every two years. The CDC laboratory follows strict quality control (QC) guidelines as mandated by CLIA. The QC process is described in Silva et al. 2013 [28]. Briefly, each group of samples is analyzed alongside QC materials and reagent blanks to ensure the accuracy and reliability of the data. The analysis of de-identified specimens at the CDC laboratory was determined not to constitute engagement in human subjects research.

### Data analysis

#### Wristband and urine data processing.

Phthalate concentrations in wristbands were log-transformed (log_2_ μg/g wristband) because of non-normal distributions. Specific gravity correction and natural-log transformation were applied to phthalate metabolite concentrations in urine (ng/mL) to adjust for urine dilution and non-normal distributions. Specific gravity-corrected metabolite concentrations were calculated with the following equation: *Pc* = *P* × [(*SG_m_* − 1)/(*SG* − 1)] where Pc is the specific gravity–corrected metabolite concentration (in ng/mL), P is the measured urinary metabolite concentration (in ng/mL), SG_m_ is the mean specific gravity of the study population (1.0156), and SG is the specific gravity of the individual urine sample [31].

### Statistical analysis.

Phthalate biomarker urine concentrations below the LOD were replaced with the $\text{LOD}/\sqrt{2}$, and phthalate concentrations in wristbands with LOD/2 [32]. Only those paired parent phthalates and urinary metabolites detected in at least 70% of samples were included in this analysis (Table 3). The molar sums of DEHP, DEHTP, DiBP, and DnBP metabolites (∑DEHP, ∑DEHTP, ∑DiBP, ∑DnBP, respectively) were calculated by dividing each corresponding metabolite concentration by its molecular weight and then summing. While ingested BBzP is excreted as both MBzP and MBP in the urine, only 6% is excreted as MBP, so MBzP was considered the primary BBzP metabolite for this analysis [33]. We include the relationship between BBzP and MBP, as well as the molar sum of MBP and MBzP, in the supplementary material (Table S4). All statistical quantities and models were calculated and fitted to each pair of parent phthalates in wristbands and phthalate metabolites in urine.

Linear regression models were fit to the data to quantify the linear relationship between urine and wristband phthalate measurements. Linear regression models were fit using the log2-adjusted wristband and ln-adjusted urinary concentrations.

K-means clustering was used to group participants into common exposure marker profiles based on the two exposure assessment tools. K-means clustering is an unsupervised clustering technique that has been used in mixture analyses to group individuals with similar exposure profiles into clusters [34–36]. Briefly, the k-means algorithm works by first randomly selecting a starting point (a centroid, or *k*) within the exposure matrix (i.e., a centroid in the CCCEH data would have values for each phthalate). The number of centroids, *k*, is pre-determined by the user. Centroid locations are recalculated based on the cluster mean, aiming to minimize the Euclidean distance between the overall cluster mean and each cluster member.

K-means clustering was applied to the wristband and urinary metabolite data sets separately. Again, only phthalates with parent compounds detected in wristbands and corresponding metabolites detected in urine were used to generate exposure marker profiles. To ensure comparability between the exposure marker profiles generated using wristband and urinary biomarker data, if a parent chemical had multiple metabolites, only the molar sum of those metabolites was used to generate the profiles. All data were first scaled and centered to avoid very large or small values from influencing the clustering solution. We selected *k* = 3 clusters for both wristbands and urine analyses because this sufficiently minimized within-cluster variation and yielded exposure profiles with more than 1 participant per cluster. This process was iterated up to 20 times until convergence under 100 random starting locations. This analysis was performed using the ‘kmeans’ function from the ‘stats’ package in R. All statistical analyses were conducted using the statistical software R, version 4.4.1 [37].

## RESULTS

### Phthalates in wristbands

DEHP, DEHTP, BBzP, DnBP, DEP, and DiBP were detected in 100%, 100%, 96.3%, 96.3%, 74.1%, and 100% of wristbands, respectively (Table 2). We collected information on an additional 25 chemicals in wristbands, and of the total 31 chemicals measured, 13 were detected at least once. DEHP, DEHTP, DiBP, and two non-phthalates, namely di-2-ethylhexyl adipate, and tris-2-ethylhexyl trimellitate, were detected the most frequently (Table S2).

### Phthalate and non-phthalate metabolites in urine

All 18 urinary phthalate and phthalate substitute metabolites measured, including two metabolites of DINCH, were detected in at least one sample (Table S3). Of these, 12 metabolites corresponded to six phthalates detected in the wristbands: MBP and MHBP (metabolites of DnBP); MHiBP and MiBP (metabolites of DiBP); MEP (metabolite of DEP); MEHP, MECPP, MEHHP, and MEOHP (metabolites of DEHP); MBzP (metabolite of BBzP); and MECPTP and MEHHTP (metabolites of DEHTP) (Table 3). All 12 metabolites were detected in more than 70% of samples.

### Quantifying associations between parent phthalates and phthalate metabolites

#### Linear relationships.

**Fig. 1:**
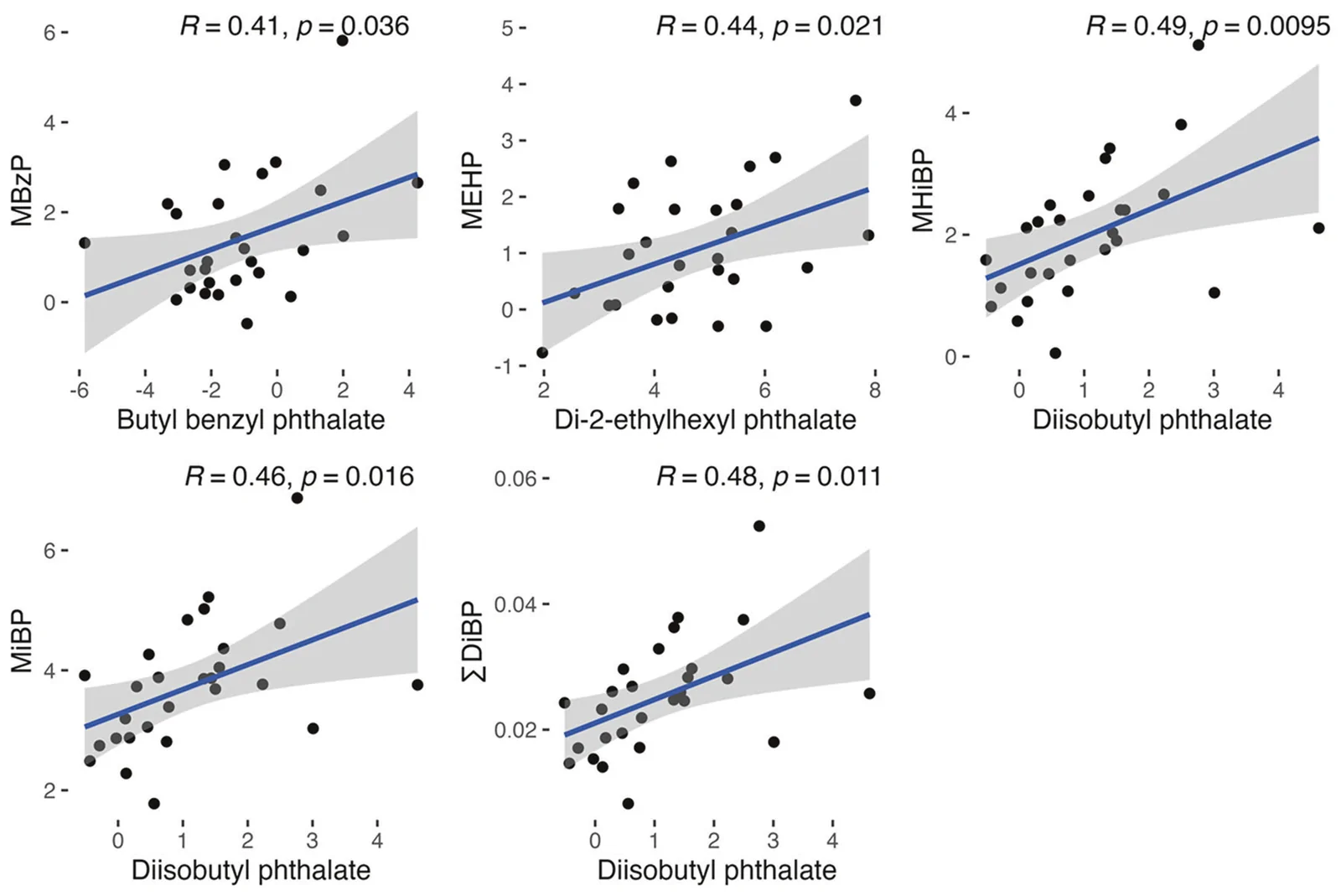
Significant linear relationships between phthalates in wristbands and their respective urinary biomarkers. From top left: BBzP and MBZP, DEHP and MEHP, DiBP and MHiBP, DiBP and MiBP, and DiBP and the molar sum of MHiBP and MiBP are presented. Parent phthalate concentrations in wristbands (μg/g) are on the x-axis and respective specific gravity-adjusted urinary metabolite concentrations (ng/mL) are on the y-axis.

Figure 1 displays the scatterplots corresponding to the five statistically significant linear relationships between parent phthalates in wristbands and corresponding metabolites in urine. BBzP was significantly positively associated with its urinary metabolite MBzP. DEHP was only statistically significantly and positively associated with one of its urinary metabolites, MEHP. DiBP was significantly associated with both of its urinary metabolites, MHiBP and MiBP, as well as ∑DiBP. While BBzP, DEHP, and DiBP had statistically significant relationships with at least one of their respective urinary metabolites, all associations were of moderate strength, with Pearson’s correlation coefficients between 0.41 and 0.49. DEHTP levels in wristbands were not associated with their urinary metabolites. All regressions between parent phthalates and respective urinary metabolites are presented in Figure S1.

#### Profiles generated from wristband and urinary biomarker data.

We explored if the wristband phthalate or urinary phthalate metabolite data generated similar concentration profiles to understand if these two exposure assessment tools reflect similar information regarding phthalate exposure.

**Fig. 2:**
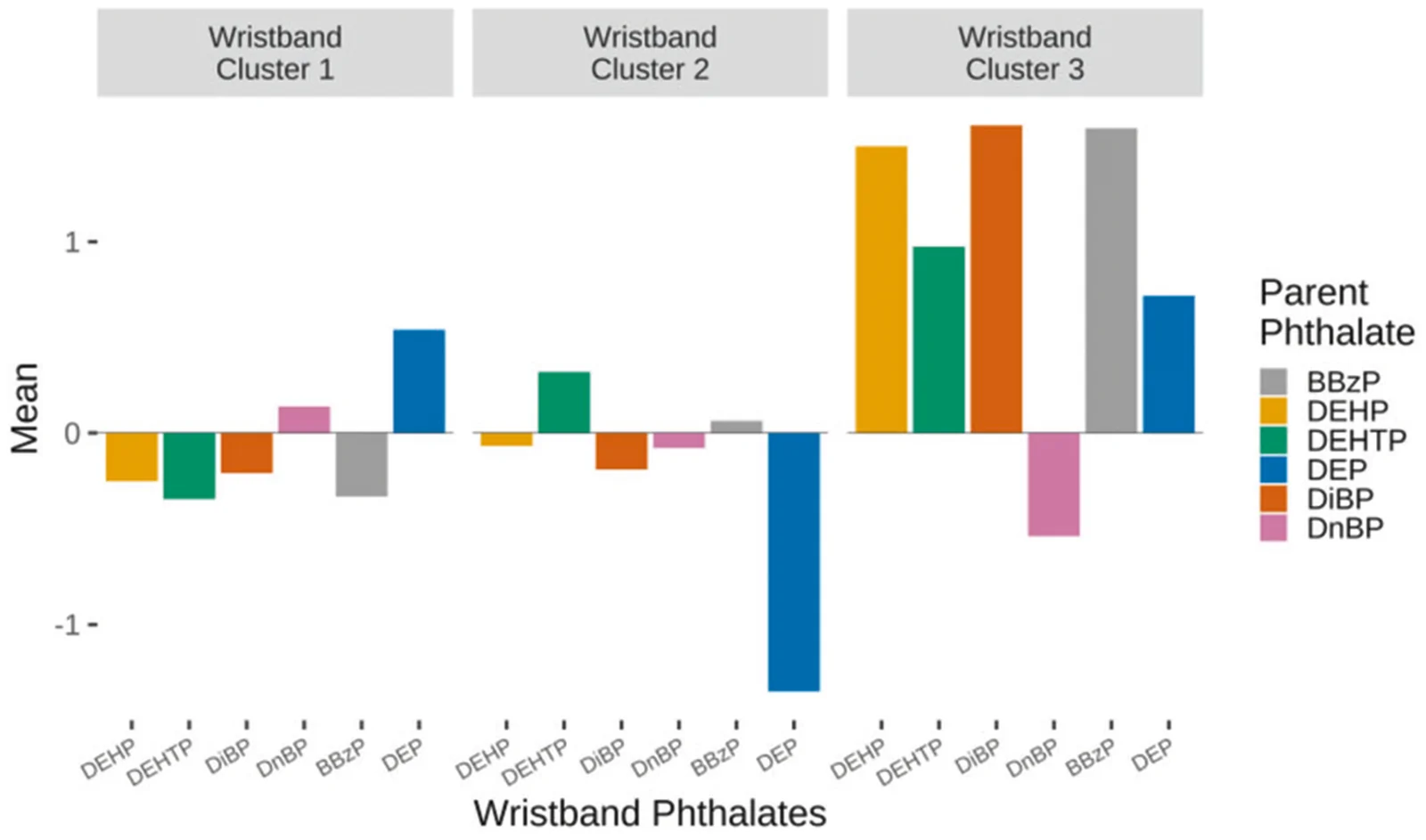
Exposure profiles generated by applying k-means clustering to the parent phthalates captured in wristbands. Wristband Cluster 1 included 16 participants, Wristband Cluster 2 included 8 participants, and 3 participants were included in Cluster 3. The x-axis displays the parent phthalate name and the y-axis displays the mean expression of the phthalate in the cluster.

The profiles generated by applying k-means clustering to the parent phthalates captured in wristbands are depicted in Fig. 2. Wristband Cluster 1 resulted in participants grouped together with profiles dominated by DEP and DnBP. Wristband Cluster 2 included participants with profiles mainly driven by DEHTP and BBzP. Wristband Cluster 3 included participants with high levels of all parent phthalates, except for DnBP.

**Fig. 3:**
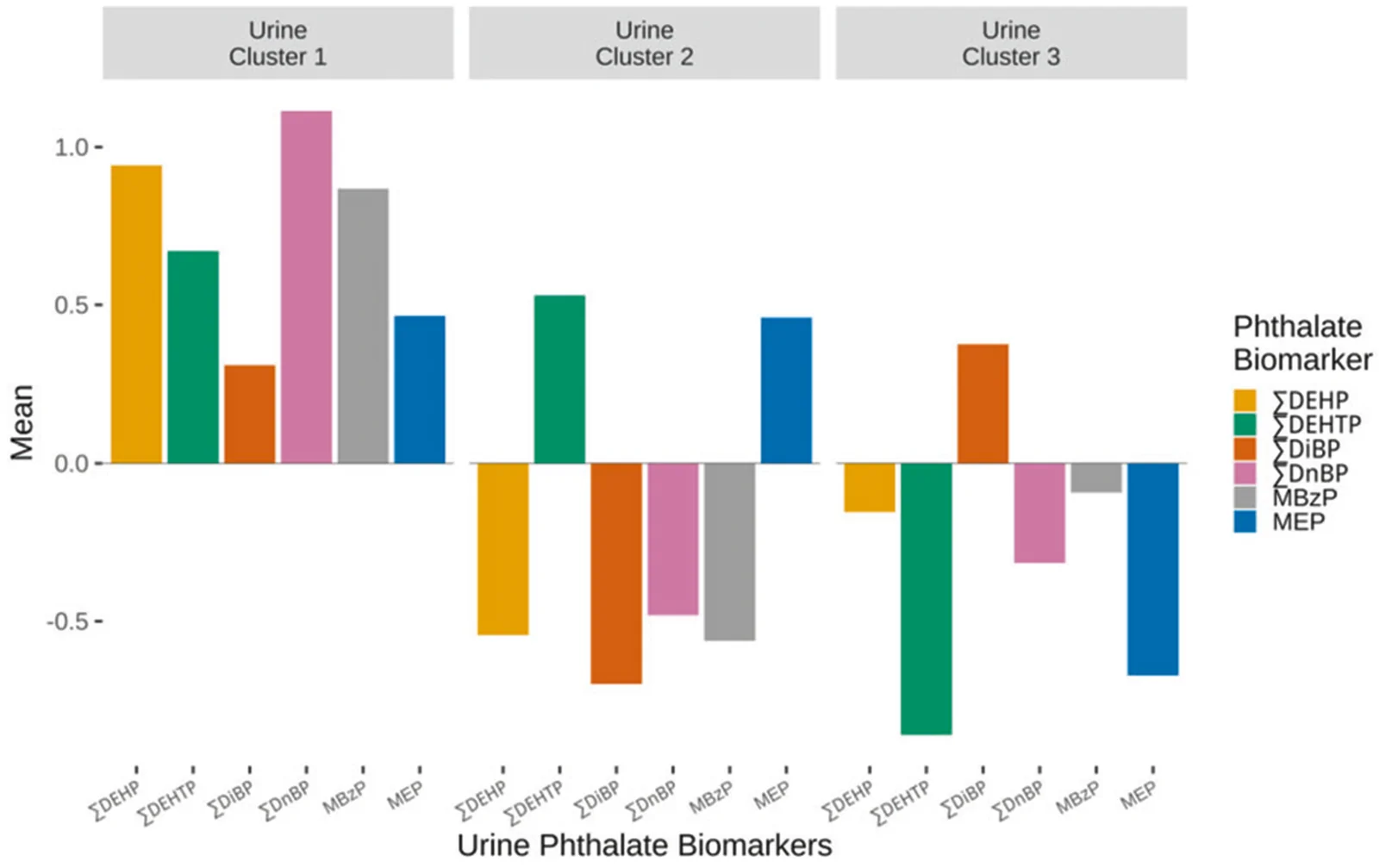
Exposure biomarker profiles generated by applying k-means clustering to the urinary phthalate metabolites captured in urine. Urine Clusters 1, 2, and 3 included 7, 9, and 11 participants respectively. The x-axis displays the phthalate metabolite name and the y-axis displays the mean expression of the phthalate metabolite in the cluster.

The profiles generated by applying k-means clustering to the urinary phthalate metabolite data are depicted in Fig. 3. Urine Cluster 1 included participants with high concentrations of all urinary phthalate metabolites measured. Urine Cluster 2 included participants with high concentrations of DEHTP metabolites and MEP, the metabolite of DEP. Urine Cluster 3 included participants recently exposed to DiBP.

**Fig. 4:**
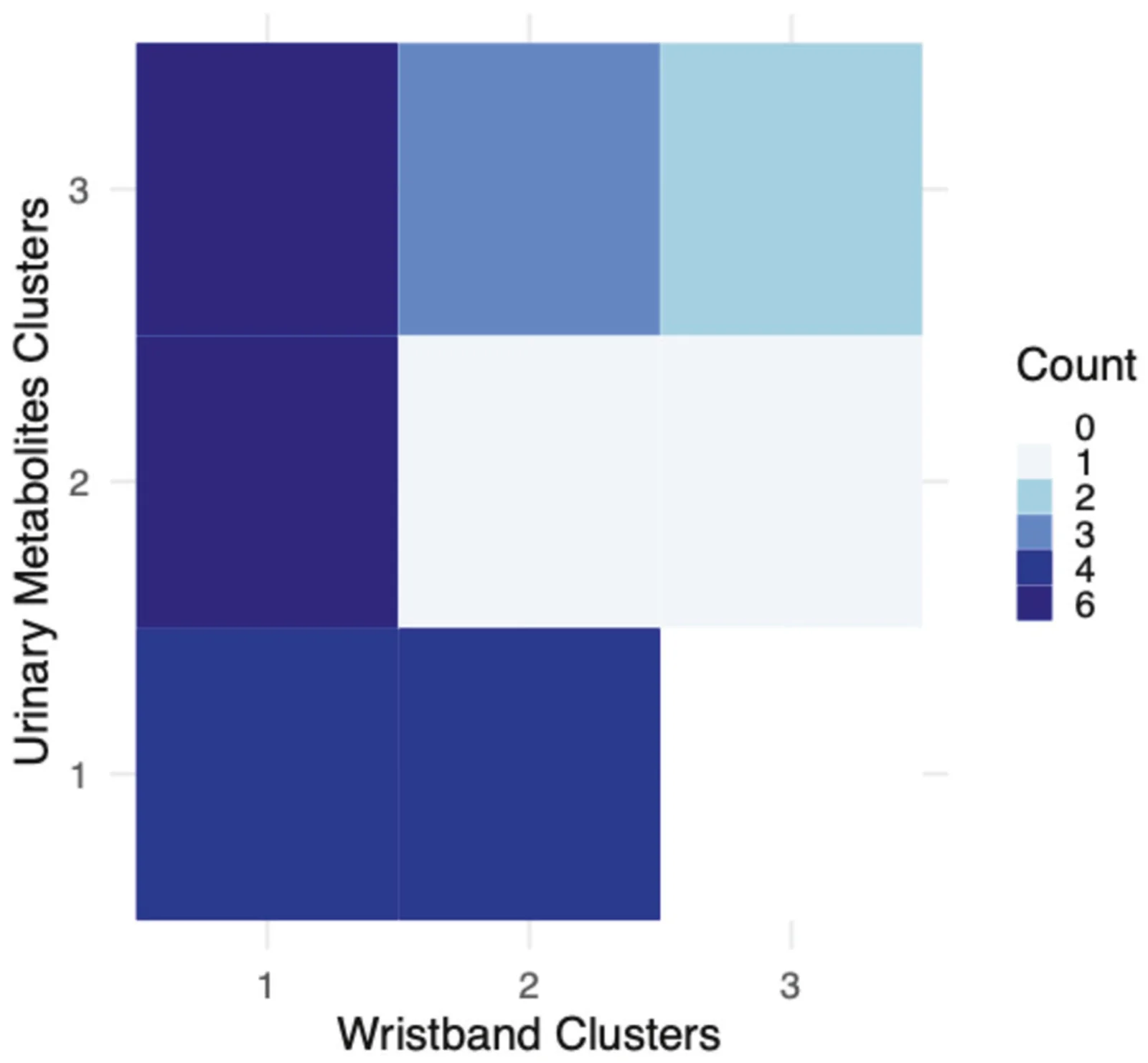
Heat map depicting participant cluster assignment overlap between the k-means clusters generated from phthalate urinary metabolite concentrations and parent compounds captured in wristbands. Wristband Clusters 1, 2, and 3 are presented on the x-axis, and Urine Clusters 1, 2, and 3 are presented on the y-axis. If there are no participants that overlap between wristband and urine cluster assignment, the corresponding color is white. The darkest blue color corresponds to the highest number of participant cluster assignment overlap (6).

When comparing individual cluster assignments across these two exposure assessment tools, we see that the highest assignment overlap between urinary phthalate metabolite concentration profiles and profiles based on phthalates captured in wristbands was for participants assigned Wristband Cluster 1 with Urine Clusters 2 or 3 (Fig. 4). Wristband Cluster 1 included 16 participants with wristband profiles dominated by DEP and DnBP. Six of these participants were clustered in Urine Cluster 3 with profiles dominated by DiBP metabolites. Six other participants were also assigned to Urine Cluster 2, with profiles dominated by DEHTP metabolites and MEP. These clustering results indicate dissimilarities in exposure marker profiles generated by the exposure assessment tool.

## DISCUSSION

In this study, we found significant linear relationships between parent phthalates detected in wristbands (DEHP, BBzP, and DiBP) and their urinary metabolites (MEHP; MBZP; MHiBP, MiBP, respectively). Significant relationships between these phthalates and their metabolites suggest that both urine biomarker concentrations and phthalate levels in wristbands can reflect personal exposure to phthalates. However, levels of many phthalates detected in wristbands were not significantly correlated with their metabolite’s urinary concentrations. For example, the replacement phthalate DEHTP was not significantly correlated with its metabolites in urine despite being detected in 100% of wristband and urine samples. These differences in exposure assessment tools are not wholly unexpected, as wristbands and urine matrices may reflect personal exposure via different pathways. Wristbands preferentially capture exposures from the air and skin of the wearer, reflecting dermal and inhalation exposure routes [15, 38]. On the other hand, urine reflects exposure from all sources and routes [8]. Urinary concentrations also reflect inter-individual variability, like physiological or metabolic differences that are not reflected in wristband parent chemical levels [39, 40]. For example, we found that DEHP was only significantly positively associated with one of its metabolites, MEHP, which could be explained in part due to differences in an individual’s ability to metabolize DEHP. Hydrolysis of DEHP to MEHP is the first step during detoxification [41]. We may not have detected a significant relationship between the secondary metabolites of DEHP because of inter-individual variability in the efficiency of oxidation of MEHP.

Because LMW phthalates like DEP, DnBP, and DiBP are used in personal care products directly applied to the skin [42, 43], exposure could be captured using wristband and urine matrices since these chemicals would be absorbed through the skin and subsequently excreted in urine. Indeed, we detected a strong relationship between DiBP levels detected in wristbands and their metabolites in urine. Yet, we did not detect significant relationships for DEP or DnBP, nor were they detected in every wristband sample, which could imply the dermal absorption and inhalation exposure routes for these phthalates are not effectively captured by the wristbands. Conversely, because HMW phthalates, like BBzP, DEHP, and DEHTP, are primarily used as plasticizers in PVC applications, like in food packaging [44–46], a majority of exposure is likely through diet and thus captured primarily via urine biomarkers. This may explain the primarily non-significant relationships between wristband DEHP and DEHTP levels and their metabolite concentrations in urine. However, these HMW phthalates are also found in PVC-based building materials like flooring, wallpaper, and paints from which they are released into the air and can settle into dust in the home, where people can be exposed via inhalation and direct contact [47, 48].

Furthermore, when comparing profiles generated from wristband data versus urine biomarker data, k-means clustering revealed distinct profiles depending on the exposure assessment tool used. For example, urinary biomarkers identified three groups of participants: those with a profile dominated by ∑DEHTP and MEP, those with higher mean concentrations of all metabolites detected, and one group dominated by ∑DiBP. Whereas wristbands identified profiles dominated by DnBP and DEP, DEHTP and BBzP, and a final group dominated by all phthalates measured except for DnBP. We also compared participant assignment overlap between these exposure assessment tools and found that the highest assignment overlaps were for participants assigned either to Urine Cluster 3 (∑DiBP) or 2 (∑DEHTP and MEP) based on urinary metabolite data and Wristband Cluster 1 (DnBP and DEP) based on wristband data. Therefore, our results suggest that the hypothesized routes of exposure to phthalates should be considered when selecting an exposure assessment tool because wristband and urine matrices capture the routes of exposure at different resolutions. Wristbands, for example, may not be the most effective personal exposure assessment method when considering phthalate exposures through diet. However, because wristbands preferentially capture the understudied inhalation and dermal exposure routes, their use could fill the gap in our understanding of the contribution and importance of these routes of exposure. [49, 50].

A study by Hammel et al. compared wristbands worn for seven days and 48-hour pooled urine samples to evaluate children’s exposure to phthalates and found similar results to ours [23]. They reported that BBzP and DiBP concentrations in wristbands were significantly positively correlated with their urinary metabolites, whereas DEHP, DnBP, and DEHTP were not. However, they found a significant positive correlation between DEP levels in wristbands and MEP concentrations in urine, whereas we did not detect a significant relationship. This discrepancy may be due in part to the larger sample size, longer timeframe that the children wore the wristbands, as well as the pharmacokinetic differences between adult and pediatric populations [51]. A small pilot study of Belgian adults also found that only the estimated dermal absorption of DiBP via wristbands worn for seven days was significantly positively correlated with the estimated daily intake of DiBP based on urinary metabolite data from four morning urine samples [20]. Our results therefore align with previous literature despite the shorter time frame our participants wore wristbands, and further highlight that, among other factors, one must understand the specific characteristics of the chemicals under study when selecting an exposure assessment method and interpreting the resulting data.

This study is not without limitations. First, Fair Start study participants may not be representative of the U.S. adult population because they were all pregnant women in their third trimester of pregnancy, most self-identified as Hispanic, lived in a major U.S. city, and volunteered to participate in a longitudinal birth cohort study. In addition to the metabolic changes that occur during gestation, pregnant women may also change their behaviors, which could alter their phthalate exposure biomarker concentrations compared to when they are not pregnant [52, 53]. Second, we used k-means clustering, an unsupervised technique, to explore how wristbands and urine as exposure assessment tools may capture phthalate exposures in our unique population. Thus, we anticipate profiles may not resemble profiles comparing exposure assessment capabilities using wristbands or urine samples in future studies with larger sample sizes or different study populations. Third, the sample size for this pilot study is small (*n* = 27), so future research with a larger sample size is necessary to validate our study findings. Due to the relatively small sample size, we were also not powered to check for seasonal effects on phthalate (bio)marker concentrations in urine and wristbands, which may be an important predictor, as prior studies have found seasonal variations in phthalate concentrations in air and dust [54, 55]. Lastly, because urine samples were not collected until the end of the 48-hour sampling period, the first two urine samples were stored in a home refrigerator for up to 48 hours before processing. It has been documented that the stability of many of the phthalate biomarkers included in this study decreases over time when stored at 4 °C, highlighting that the measured biomarker concentrations in our study may be an underestimate [56]. Thus, we may be underestimating the strength of the relationships between wristband and urinary phthalate (bio) marker concentrations.

## CONCLUSIONS

In this pilot study, we found significant linear relationships between parent phthalates DEHP, BBzP, and DiBP in wristbands and their urinary metabolites MEHP, MBZP, MHiBP and MiBP, respectively, demonstrating that phthalate levels in wristbands can reflect personal exposure to phthalates similar to urine biomarkers. Because wristbands preferentially capture the understudied inhalation and dermal exposure routes, their use could fill the gap in our understanding of the contribution and importance of these exposure routes for phthalates and their substitutes.

## Supplementary Material

Supplementary material

The online version contains supplementary material available at https://doi.org/10.1038/s41370-026-00899-y.

## Figures and Tables

**Table 1.** Study participant demographics of the Fair Start Cohort.

Demographic Characteristics	*N* (%) (Total *N* = 27)
**Age (years)**	
Median [Min, Max]	26.5 [18.3, 42.7]
**Medicaid**	
Yes	25 (92.6%)
No	1 (3.7%)
Unknown or not reported	1 (3.7%)
**Maternal Education**	
Did not complete high school	6 (22.2%)
High school graduate	7 (25.9%)
More than high school	14 (51.9%)
**Maternal Ethnicity**	
Hispanic or Latino	25 (92.6%)
Not Hispanic or Latino	1 (3.7%)
Unknown or not reported	1 (3.7%)

**Table 2.** Summary statistics for phthalates detected in ≥70% of wristbands (μg/g).

Parent Compound	Geometric Mean (μg/g)	Instrument LOD (μg/g)	Detection Frequency (%)
Di-2-ethylhexyl phthalate	26.9	0.03	100
Di-2-ethylhexyl terephthalate	39.8	0.12	100
Butyl benzyl phthalate	0.5	0.03	96.3
Di-n-butyl phthalate	1.4	0.04	96.3
Diethyl phthalate	0.3	0.03	74.1
Di-iso-butyl phthalate	2.1	0.03	100

**Table 3.** Urinary phthalate biomarker concentrations, adjusted for specific gravity (ng/mL) for phthalate metabolites detected in ≥70% of urine samples and with corresponding parent compounds detected in wristbands.

Parent Compound	Urinary Metabolite	Geometric Mean	Instrument LOD	Detection frequency (%)
Di-2-ethylhexyl phthalate (DEHP)	MECPP	10.6	0.4	100
	MEOHP	6.3	0.2	100
	MEHHP	7.5	0.4	100
	MEHP	2.1	0.8	81.5
	ΣDEHP	0.1	-	-
Di-2-ethylhexyl terephthalate (DEHTP)	MECPTP	58.7	0.2	100
	MEHHTP	9.1	0.4	100
	ΣDEHTP	0.2	-	-
Butyl benzyl phthalate (BBzP)	MBzP	2.7	0.3	100
Di-n-butyl phthalate (DnBP)	MBP	16	0.4	100
	MHBP	1.3	0.4	96.3
	ΣDnBP	0.1	-	-
Diethyl phthalate (DEP)	MEP	41.3	1.2	100
Di-iso-butyl phthalate (DiBP)	MHiBP	4	0.4	100
	MiBP	13.2	0.8	100
	ΣDiBP	0.1	-	-

## Data Availability

The de-identified data analyzed within this study are available from the corresponding author upon request.
