# Supplementary material for: Evaluating the use of silicone wristbands and urinary biomarkers to assess personal exposure to phthalates
Source: J Expo Sci Environ Epidemiol. 2026 Apr 28;36(6):1171–8. doi: 10.1038/s41370-026-00899-y (PMC13339999; doi:10.1038/s41370-026-00899-y)
Supplement: Supplementary file 1 [file 41370_2026_899_MOESM1_ESM.docx]

**Supplementary Material**

**Evaluating the use of silicone wristbands and urinary biomarkers to assess personal exposure to phthalates**

Alana J. Ferris^1^, Kylie W. Riley^2^, Lehyla Calero^2^, Darrell Holmes^2^, Catherine Tobon^2^, Matthew Gutierrez^2^, Julianne Cook Botelho^3^, Antonia M. Calafat^3^, Maya A. Deyssenroth^1^, Kim A. Anderson^4^, Julie B. Herbstman^2^

^1^Department of Environmental Health Sciences, Mailman School of Public Health, Columbia University, New York City, NY, United States

^2^ Columbia Center for Children’s Environmental Health, Department of Environmental Health Sciences, Mailman School of Public Health, Columbia University, New York City, NY, United States

^3^Division of Laboratory Sciences, Centers for Disease Control and Prevention, Atlanta, GA, United States

^4^Environmental and Molecular Toxicology, Food Safety and Environmental Stewardship Program, Oregon State University, Corvallis, OR, United States

**Table of Contents**

**Table S 1** Wristband and urine sample collection seasons…….………….……………………....2

**Table S 2** Summary statistics for all wristband target compounds on the μg/g scale.………........3

**Table S 3** Summary statistics for all urinary target biomarkers, adjusted for specific gravity (ng/mL)……………………..……………………………………………………………………..4

**Figure S 1** Scatterplots of phthalate concentrations in wristbands plotted against urinary metabolite concentrations…………………………………………………………………………6

**Table S 4** Exploratory analysis examining the linear relationship between butyl benzyl phthalate levels in wristbands and the urinary metabolite MBP concentrations………….…………………7

|  | **Season** | | | |
| --- | --- | --- | --- | --- |
| **Years** | **Winter**  (December-February) | **Spring**  (March-May) | **Summer**  (June-August) | **Fall**  (September-November) |
| 2018-2019 | 11 | 2 | 8 | 6 |

**Table S 1.** Wristband and urine sample collection season.

| **Parent Compound** | **Geometric Mean** (μg/g) | **Instrument LOD** (μg/g) | **Detection Frequency** (%) |
| --- | --- | --- | --- |
| Di-2-butoxyethyl phthalate | - | 0.01 | 0 |
| Di-2-ethoxyethyl phthalate | - | 0.01 | 0 |
| Di-2-ethylhexyl isophthalate^1^ | - | 0.03 | 3.7 |
| **Di-2-ethylhexyl phthalate** | 26.9 | 0.03 | 100 |
| **Di-2-ethylhexyl terephthalate** | 39.8 | 0.12 | 100 |
| Di-2-methoxyethyl-phthalate | - | 0.01 | 0 |
| Di-2-propylheptyl phthalate | - | 0.01 | 0 |
| Di-4-methylpentyl phthalate | - | 0.01 | 0 |
| **Butyl benzyl phthalate** | 0.5 | 0.03 | 96.3 |
| Di-2-ethylhexyl adipate^a^ | 4.5 | 0.03 | 100 |
| **Di-n-butyl phthalate** | 1.4 | 0.04 | 96.3 |
| Di-n-hexyl phthalate | - | 0.03 | 0 |
| Di-n-nonyl phthalate | 0.1 | 0.05 | 29.6 |
| Di-n-octyl phthalate^2^ | - | 0.03 | 3.7 |
| Di-n-propyl phthalate | - | 0.03 | 0 |
| Diallyl phthalate | - | 0.01 | 0 |
| Diamyl phthalate | - | 0.01 | 0 |
| Dibenzyl phthalate | - | 0.01 | 0 |
| Diamyl phthalate | - | 0.01 | 0 |
| Dicyclohexyl phthalate | - | 0.04 | 0 |
| Didecyl phthalate | 0.1 | 0.05 | 14.8 |
| **Diethyl phthalate** | 0.3 | 0.03 | 74.1 |
| Diheptyl phthalate | - | 0.04 | 0 |
| **Diisobutyl phthalate** | 2.1 | 0.03 | 100 |
| Diundecyl phthalate | 0.02 | 0.01 | 29.6 |
| Diisopentyl phthalate | - | 0.01 | 0 |
| Diisopropyl phthalate | - | 0.01 | 0 |
| Dimethyl phthalate | - | 0.04 | 0 |
| Diphenyl isophthalate | - | 0.03 | 0 |
| Ditridecyl phthalate | - | 0.01 | 0 |
| Diisodecyl phthalate | - | 0.01 | 0 |
| Tris(2-ethylhexyl) trimellitate^a^ | 0.8 | 0.02 | 100 |

**Table S 2.** Summary statistics of all target wristband phthalates and replacements, including limits of detection (LOD; μg/g). The reporting limits here include what is calculated with real matrices and potential interferences. ^1^Di(2-ethylhexyl) isophthalate was detected once at a concentration of 1.03ug/g. ^2^Di-n-octyl phthalate was detected once at a concentration of 4.52ug/g. ^a^Non-phthalate replacement. Compounds in **bold** correspond with the paired urinary metabolites in this study.

**Table S 3.** Summary statistics for all urinary phthalate and substitute biomarkers, adjusted for specific gravity (ng/mL). Chemicals in **bold** correspond with the paired wristband analytes in this study. ^a^Phthalate substitute. Abbreviations: LOD, limits of detection.

| **Analyte name** | **Analyte code** | **LOD** (ng/mL) | **Geometric Mean** (ng/mL) | **Detection Frequency** (%) | **Parent compound** |
| --- | --- | --- | --- | --- | --- |
| **Mono-2-ethyl-5-carboxypentyl phthalate** | **MECPP** | 0.4 | 10.61 | 100 | Di-2-ethylhexyl phthalate (DEHP) |
| **Mono-2-ethyl-5-hydroxyhexyl phthalate** | **MEHHP** | 0.4 | 7.54 | 100 |  |
| **Mono-2-ethyl-5-oxohexyl phthalate** | **MEOHP** | 0.2 | 6.28 | 100 |  |
| **Mono-2-ethylhexyl phthalate** | **MEHP** | 0.8 | 2.09 | 80 |  |
| Mono-3-carboxypropyl phthalate | MCPP | 0.4 | 0.88 | 90 | Di-n-octyl phthalate (DOP) and other high molecular weight phthalates; (DBP; minor) |
| **Mono-n-butyl phthalate** | **MBP** | 0.4 | 16.05 | 100 | Di-n-butyl phthalate (DBP or DnBP) |
| **Mono-hydroxybutyl phthalate** | **MHBP** | 0.4 | 1.3 | 92.5 |  |
| **Mono-isobutyl phthalate** | **MiBP** | 0.8 | 13.2 | 100 | Di-iso-butyl phthalate (DiBP) |
| **Mono-hydroxy-isobutyl phthalate** | **MHiBP** | 0.4 | 4.0 | 100 |  |
| **Monobenzyl phthalate** | **MBzP** | 0.3 | 2.7 | 100 | Butylbenzyl phthalate (BBzP) |
| **Monoethyl phthalate** | **MEP** | 1.2 | 41.3 | 100 | Diethyl phthalate (DEP) |
| Monooxononyl phthalate | MONP | 0.4 | 1.9 | 100 | Di-isononyl phthalate (DiNP) |
| Mono carboxyisooctyl phthalate | MCOP | 0.3 | 5.2 | 100 |  |
| Mono carboxyisononyl phthalate | MCNP | 0.2 | 1.1 | 97.5 | Di-isodecyl phthalate (DiDP) |
| Cyclohexane-1,2-dicarboxylic acid, monocarboxy isooctyl ester | MCOCH | 0.5 | 0.6 | 40 | 1,2-Cyclohexane dicarboxylic acid, diisononyl ester (DINCH)^a^ |
| Cyclohexane-1,2-dicarboxylic acid, monohydroxy isononyl ester | MHiNCH | 0.4 | 1.1 | 85 |  |
| **Mono-2-ethyl-5-carboxypentyl terephthalate** | **MECPTP** | 0.2 | 58.7 | 100 | Di-2-ethylhexyl terephthalate (DEHTP) |
| **Mono-2-ethyl-5-hydrohexyl terephthalate** | **MEHHTP** | 0.4 | 9.1 | 100 |  |

**Table S 3** (continued)**.** Summary statistics for all urinary phthalate and substitute biomarkers, adjusted for specific gravity (ng/mL). Compounds in **bold** correspond with the paired wristband compounds in this study. ^a^Non-phthalate replacement. Abbreviations: LOD, limits of detection.

**Figure S 1.** Scatterplots of linear relationships between paired log2 phthalate concentrations in wristbands (μg/g) and respective specific gravity- and ln-adjusted urinary metabolite concentrations.

| **Parent compound** | **Urinary metabolite name** | **Urinary metabolite code** | **Estimate** | ***p*-value** |
| --- | --- | --- | --- | --- |
| Butyl benzyl phthalate (BBzP) | Mono-n-butyl phthalate | MBP | 0.02 | 0.87 |
|  | Monobenzyl phthalate | MBzP | 0.27 | 0.04 |
|  | Mono-n-butyl phthalate + monobenzyl phthalate | ∑BBzP | 0.001 | 0.17 |
| Butyl benzyl + Di-n-butyl phthalate | Mono-n-butyl phthalate | MBP | 0.05 | 0.5 |

**Table S 4.** Examining the linear relationship between log_2_ butyl benzyl phthalate (BBzP) levels in wristbands (μg/g) and a minor urinary metabolite, mono-n-butyl phthalate, in urine (specific gravity- and ln-adjusted concentrations), as well as the linear relationship between BBzP and the sum of its urinary metabolites. We also explore the linear relationship between the sum of BBzP and Di-n-butyl phthalate (DnBP) and MBP, since both are parent compounds of MBP.
